# Supplementary figures and images for: Factors influencing the performance of cardiopulmonary resuscitation by lay rescuers: A qualitative study based on the Theory of Planned Behavior
Source: PLoS One. 2025 Jul 23;20(7):e0327439. doi: 10.1371/journal.pone.0327439 (PMC12286394; doi:10.1371/journal.pone.0327439)

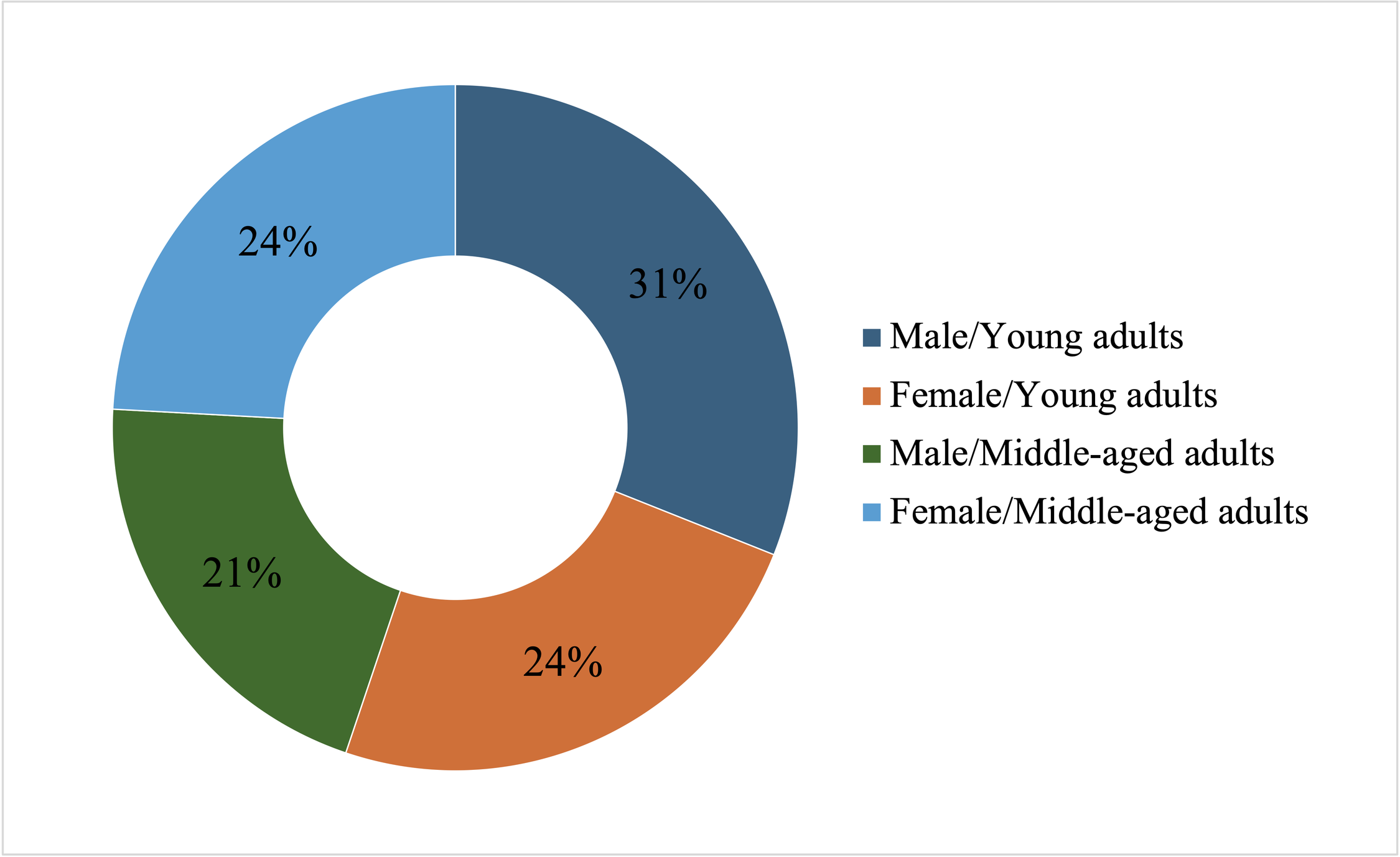

Supplement: S1 Fig — (TIF) [file pone.0327439.s001.tif]
