# Supplementary material for: Factors influencing the performance of cardiopulmonary resuscitation by lay rescuers: A qualitative study based on the Theory of Planned Behavior
Source: PLoS One. 2025 Jul 23;20(7):e0327439. doi: 10.1371/journal.pone.0327439 (PMC12286394; doi:10.1371/journal.pone.0327439)
Supplement: Attachment — Submitted filename: PONE-D-24-25699_reviewer.pdf [file pone.0327439.s002.pdf]

PLOS ONE

Factors influencing the performance of cardiopulmonary resuscitation by lay rescuers: a qualitative study based on the Theory of Planned Behavior

--Manuscript Draft--

**Manuscript Number:**	PONE-D-24-25699
**Article Type:**	Research Article
**Full Title:**	Factors influencing the performance of cardiopulmonary resuscitation by lay rescuers: a qualitative study based on the Theory of Planned Behavior
**Short Title:**	Factors affecting bystander cardiopulmonary resuscitation
**Corresponding Author:**	Liang LIU<br>The Second Xiangya Hospital of Central South University Department of Nephrology<br>Changsha, CHINA
**Keywords:**	cardiopulmonary resuscitation; Behavioral Intentions; theory of planned behavior; Lay Rescuers
**Abstract:**	Abstracts<br>Aims: The paper aims to explore the reasons for the current low implementation rate of cardiopulmonary resuscitation (CPR) among lay rescuers, and the factors affecting their intention to perform CPR and the conversion from intention to behavior, so as to propose measures to increase the rate of bystanders implementing CPR.<br>Methods: This was a qualitative study using purposive sampling. Semi-structured interviews were conducted during the period from 1/January/2022 to 20/May/2022.The research data was processed by Nvivo software and analysed by thematic analysis.<br>Results: A total of 29 lay rescuers participated in the study. Eleven themes have been identified in the four concepts of the Theory of Planned Behavior (TPB). For attitudes, there were two themes with two sub-themes: (1) emotional resistance to the death event; (2) awareness of the benefits of rescuing (possible external rewards, ability to achieve intrinsic self-worth). For perceived behavioral norms, three themes were identified: (1) the bystander effect; (2) encouraging effect of peers; (3) personal motivation brought about by the career. For perceived behavioral control, three themes and two sub-themes emerged: (1) consideration of possible consequences (getting into various legal disputes, fear of being infected with a disease); (2) the dual effect of public opinion pressure; (3) low self-efficacy. And for actual control, three themes and two sub-themes were identified: (1) acquisition of CPR skills; (2) support or obstacles at the scene (support from public facilities or procedures, reduced sense of responsibility at special locations); (3) impact of patient status.<br>Conclusions: Many new factors have been identified, which influence public intention to perform CPR and conversion from intention to behavior. Trainers should be aware of these factors in order to improve the CPR training content, which would increase the public's willingness to perform CPR. Meanwhile, they should increase the existing CPR resources, including the formulation, refinement and promotion of relevant laws and policies, the improvement of public emergency facilities, and the popularization of CPR skills. By doing these, the public's willingness to perform CPR can be promoted and the practical barriers to its implementation can be reduced.
**Order of Authors:**	ZHONG Zhikang, RN M.D.
	LI Weiwei, RN M.D.
	ZHOU Lin, M.D.
	Liang LIU, RN M.D.
**Additional Information:**	
**Question**	**Response**
**Financial Disclosure**<br><br>Enter a financial disclosure statement that describes the sources of funding for the	Yes

work included in this submission. Review the submission guidelines for detailed requirements. View published research articles from *PLOS ONE* for specific examples.<br><br>This statement is required for submission and **will appear in the published article** if the submission is accepted. Please make sure it is accurate.<br><br>**Funded studies**<br>Enter a statement with the following details:<br>• Initials of the authors who received each award<br>• Grant numbers awarded to each author<br>• The full name of each funder<br>• URL of each funder website<br>• Did the sponsors or funders play any role in the study design, data collection and analysis, decision to publish, or preparation of the manuscript?<br><br>Did you receive funding for this work?	
Please add funding details.<br>as follow-up to "**Financial Disclosure**<br><br>Enter a financial disclosure statement that describes the sources of funding for the work included in this submission. Review the submission guidelines for detailed requirements. View published research articles from *PLOS ONE* for specific examples.<br><br>This statement is required for submission and **will appear in the published article** if the submission is accepted. Please make sure it is accurate.	This study was supported by a scientific research project from the Hunan Provincial Health Commission (Project No. D202303056656), which was awarded the project to Ms ZHOU, which provided financial support for the subsequent APC.

Powered by Editorial Manager® and ProduXion Manager® from Aries Systems Corporation

**Funded studies**<br>Enter a statement with the following details:<br>• Initials of the authors who received each award<br>• Grant numbers awarded to each author<br>• The full name of each funder<br>• URL of each funder website<br>• Did the sponsors or funders play any role in the study design, data collection and analysis, decision to publish, or preparation of the manuscript?<br><br>Did you receive funding for this work?"	
Please select the country of your main research funder (please select carefully as in some cases this is used in fee calculation).<br>as follow-up to "**Financial Disclosure**<br><br>Enter a financial disclosure statement that describes the sources of funding for the work included in this submission. Review the submission guidelines for detailed requirements. View published research articles from *PLOS ONE* for specific examples.<br><br>This statement is required for submission and **will appear in the published article** if the submission is accepted. Please make sure it is accurate.<br><br>**Funded studies**<br>Enter a statement with the following details:<br>• Initials of the authors who received each award<br>• Grant numbers awarded to each author<br>• The full name of each funder<br>• URL of each funder website<br>• Did the sponsors or funders play any role in the study design, data collection and analysis, decision to publish, or preparation of the manuscript?<br><br>Did you receive funding for this work?"	CHINA - CN
**Competing Interests**	The authors have declared that no competing interests exist.

Use the instructions below to enter a competing interest statement for this submission. On behalf of all authors, disclose any competing interests that could be perceived to bias this work—acknowledging all financial support and any other relevant financial or non-financial competing interests.<br><br>This statement is required for submission and **will appear in the published article** if the submission is accepted. Please make sure it is accurate and that any funding sources listed in your Funding Information later in the submission form are also declared in your Financial Disclosure statement.<br><br>View published research articles from *PLOS ONE* for specific examples.<br><br>**NO authors have competing interests**<br><br>Enter: *The authors have declared that no competing interests exist.*<br><br>**Authors with competing interests**<br><br>Enter competing interest details beginning with this statement:<br><br>*I have read the journal's policy and the authors of this manuscript have the following competing interests: [insert competing interests here]*<br><br>* typeset	
**Ethics Statement**<br><br>Enter an ethics statement for this submission. This statement is required if the study involved:<br><br>• Human participants<br>• Human specimens or tissue<br>• Vertebrate animals or cephalopods<br>• Vertebrate embryos or tissues	N/A

• Field research<br><br>Write "N/A" if the submission does not require an ethics statement.<br><br>General guidance is provided below. Consult the submission guidelines for detailed instructions. **Make sure that all information entered here is included in the Methods section of the manuscript.**	

Powered by Editorial Manager® and ProduXion Manager® from Aries Systems Corporation

**Format for specific study types**<br><br>**Human Subject Research (involving human participants and/or tissue)**<br>• Give the name of the institutional review board or ethics committee that approved the study<br>• Include the approval number and/or a statement indicating approval of this research<br>• Indicate the form of consent obtained (written/oral) or the reason that consent was not obtained (e.g. the data were analyzed anonymously)<br><br>**Animal Research (involving vertebrate animals, embryos or tissues)**<br>• Provide the name of the Institutional Animal Care and Use Committee (IACUC) or other relevant ethics board that reviewed the study protocol, and indicate whether they approved this research or granted a formal waiver of ethical approval<br>• Include an approval number if one was obtained<br>• If the study involved *non-human primates*, add *additional details* about animal welfare and steps taken to ameliorate suffering<br>• If anesthesia, euthanasia, or any kind of animal sacrifice is part of the study, include briefly which substances and/or methods were applied<br><br>**Field Research**<br><br>Include the following details if this study involves the collection of plant, animal, or other materials from a natural setting:<br>• Field permit number<br>• Name of the institution or relevant body that granted permission	
**Data Availability**<br><br>Authors are required to make all data underlying the findings described fully available, without restriction, and from the time of publication. PLOS allows rare exceptions to address legal and ethical concerns. See the PLOS Data Policy and FAQ for detailed information.	Yes - all data are fully available without restriction

A Data Availability Statement describing where the data can be found is required at submission. Your answers to this question constitute the Data Availability Statement and **will be published in the article**, if accepted.<br><br>**Important:** Stating 'data available on request from the author' is not sufficient. If your data are only available upon request, select 'No' for the first question and explain your exceptional situation in the text box.<br><br>Do the authors confirm that all data underlying the findings described in their manuscript are fully available without restriction?	
**Describe where the data may be found in full sentences. If you are copying our sample text, replace any instances of XXX with the appropriate details.**<br><br>• If the data are **held or will be held in a public repository**, include URLs, accession numbers or DOIs. If this information will only be available after acceptance, indicate this by ticking the box below. For example: *All XXX files are available from the XXX database (accession number(s) XXX, XXX.).*<br>• If the data are all contained **within the manuscript and/or Supporting Information files**, enter the following: *All relevant data are within the manuscript and its Supporting Information files.*<br>• If neither of these applies but you are able to provide **details of access elsewhere**, with or without limitations, please do so. For example:<br><br>*Data cannot be shared publicly because of [XXX]. Data are available from the XXX Institutional Data Access / Ethics Committee (contact via XXX) for researchers who meet the criteria for access to confidential data.*<br><br>*The data underlying the results presented in the study are available from (include the name of the third party*	All relevant data are within the manuscript and its Supporting Information files.

and contact information or URL).<br>• This text is appropriate if the data are owned by a third party and authors do not have permission to share the data.<br><br>* typeset	
Additional data availability information:	

Powered by Editorial Manager® and ProduXion Manager® from Aries Systems Corporation

Manuscript Click here to access/download;Manuscript;manuscript-1.docx

Factors influencing the performance of cardiopulmonary resuscitation by lay rescuers: a qualitative study based on the Theory of Planned Behavior

ZHONG Zhikang[1];LI Weiwei[2];ZHOU Lin[3]; LIU Liang[4]

1 RN M.D. Department of Nephrology, The Second Xiangya Hospital, Central South University, 139 Renmin Middle Road, Changsha, Hunan, China, 410011. E-mail:1245928939@qq.com，ORCID: https://orcid.org/0009-0002-2667-4102

2. RN M.D. Department of Geriatrics, People's Hospital of Wuhan University, 99 Zhangzhidong Road, Wuhan, Hubei, China. E-mail:450008333@qq.com

3. RN M.D. Department of Nephrology, The Second Xiangya Hospital, Central South University, 139 Renmin Middle Road, Changsha, Hunan, China, 410011. E-mail: 517288621@qq.com

4. RN M.D. Department of Nephrology, The Second Xiangya Hospital, Central South University, 139 Renmin Middle Road, Changsha, Hunan, China, 410011. E-mail:507242@csu.edu.cn，ORCID：https://orcid.org/0000-0003-3817-1756

Corresponding author：LIU Liang

E-mail:507242@csu.edu.cn，ORCID：https://orcid.org/0000-0003-3817-1756

Abstracts

Aims: The paper aims to explore the reasons for the current low implementation rate of cardiopulmonary resuscitation (CPR) among lay rescuers, and the factors affecting their intention to perform CPR and the conversion from intention to behavior, so as to propose measures to increase the rate of bystanders implementing CPR.

Methods: This was a qualitative study using purposive sampling. Semi-structured interviews were conducted during the period from 1/January/2022 to 20/May/2022.The research data was processed by Nvivo software and analysed by thematic analysis.

Results: A total of 29 lay rescuers participated in the study. Eleven themes have been identified in the four concepts of the Theory of Planned Behavior (TPB). For attitudes, there were two themes with two sub-themes: (1) emotional resistance to the death event; (2) awareness of the benefits of rescuing (possible external rewards, ability to achieve intrinsic self-worth). For perceived behavioral norms, three themes were identified: (1) the bystander effect; (2) encouraging effect of peers; (3) personal motivation brought about by the career. For perceived behavioral control, three themes and two sub-themes emerged: (1) consideration of possible consequences (getting into various legal disputes, fear of being infected with a disease); (2) the dual effect of public opinion pressure; (3) low self-efficacy. And for actual control, three themes and two sub-themes were identified: (1) acquisition of CPR skills; (2) support or obstacles at the scene (support from public facilities or procedures, reduced sense of responsibility at special locations); (3) impact of patient status.

Conclusions: Many new factors have been identified, which influence public intention to perform CPR and conversion from intention to behavior. Trainers should be aware of these

factors in order to improve the CPR training content, which would increase the public's willingness to perform CPR. Meanwhile, they should increase the existing CPR resources, including the formulation, refinement and promotion of relevant laws and policies, the improvement of public emergency facilities, and the popularization of CPR skills. By doing these, the public's willingness to perform CPR can be promoted and the practical barriers to its implementation can be reduced.

Keywords: Cardiopulmonary Resuscitation; Behavioral Intentions; Theory of Planned Behavior; Lay Rescuers

1 Introduction

Out-of-hospital cardiac arrest (OHCA) is one of the global leading causes for death[1]. According to *the 2020 American Heart Association Guidelines for Cardiopulmonary Resuscitation and Emergency Cardiovascular Care* (hereinafter referred to as *The AHA Guidelines*), as of 2015, approximately 350,000 people in the U.S. experienced OHCA via Emergency Medical Service (EMS). *The China Cardiovascular Health and Disease Report 2021* showed that the number of cardiovascular disease patients in China was about 330 million; and that of sudden cardiac deaths was as high as 550,000 each year, with more than 60% of them occurring outside of healthcare facilities, and the out-of-hospital survival rate of less than 1%[2]. In 2020, the survival rate of OHCA patients in China was only 1.2%, and the rate of good neurological prognosis was 0.8 %[3]. The chances of survival in OHCA patients are more than doubled by rapid cardiopulmonary resuscitation (CPR) after OHCA[4]. Studies have shown that 4 minutes after cardiac arrest is the prime response time for resuscitation, but the actual median response time for emergency medical services in mainland China was 12 minutes[3]. This suggested that it is required for lay rescuers to rapidly recognize cardiac arrest and initiate CPR. Therefore, improving the CPR implementation rate of lay rescuers is particularly important. However, the rate in China is from 2.8% to 11.4%[5], far lower than that of other developed countries in the world[6, 7].

In addition, the main measures to increase CPR implementation rate are CPR skills promotion and training, but current training focuses on transferring knowledge and

skills to individuals, which may not be effective in increasing CPR implementation intentions. A study by Robert Swor and colleagues showed that CPR participants still had difficulty implementing CPR after training, resulting in inadequate CPR implementation[8]. After a large-scale CPR education campaign across the United States, only 30-40% of OHCA patients received CPR[9]. *The AHA Guidelines* also emphasised the importance of improving CPR intentions[10]. Intention is the perceived probability of an individual to perform a specific task with a specific intent, and good intention is effective in promoting behavior. For example, *The AHA Guidelines* recommended that lay rescuers use only chest compressions[11]. This avoids the potential for disease transmission from mouth-to-mouth resuscitation and therefore improves their willingness to perform resuscitation, effectively increasing CPR implementation rate[12]. Meanwhile, research has shown that when an individual performs an event, his or her positive intention does not necessarily mean the occurrence of behavior, and there is no consistency between the individual's intention and the occurrence of behavior[9]. A strong intention to perform can be converted into behavior in a practical setting without excessive barriers. Therefore, the public CPR implementation rate can be effectively improved by identifying the factors that influence lay rescuers' intention to implement CPR and the obstacles to the conversion of intention into behavior,.

Theory of Planned Behavior (TPB) can explain the complex relationship between human behavior and intention. Scholar Ajzen[13] proposed TPB in 1985, which then has been widely used to explain and study a number of human behaviors[14, 15]. It has

also been applied effectively in health care fields such as hand hygiene and diabetes management[16-18]. TPB suggested that an individual's behavior is influenced not only by his or her attitudes and experiences, but also by perceived social pressures to take (or not to take) action. The model is a good predictor of the factors that influence an individual's behavioral intentions when faced with a health-related event[19]. The theory also stated that intention acts as an antecedent to influencing behavior, and thus moderating intention can effectively influence intervention behavior. However, whether or not the behavior is ultimately implemented still depends on influencing factors between intention and behavior, and the positive intention does not necessarily mean the occurrence of behavior. According to this theory, the occurrence of a particular behavior depends on the strength of an individual's intention, which is influenced not only by knowledge and beliefs, but also by multidimensional factors such as the individual's preconceptions of consequences, self-control, and real-world obstacles. Identifying the factors that enhance or diminish lay rescuers' intention to perform CPR can effectively improve public intention and the conversion from intention to behavior. Therefore, it is necessary to explore these factors, so as to improve the implementation rate of CPR among the public.

Therefore, this study used semi-structured interviews and a TPB-based approach to further explore the risk or protective factors for lay rescuers' intention to perform CPR. Based on this information, clinical and training organisations can develop measures to improve CPR implementation rates among the public.

2 Methods

2.1 Design

This is a qualitative study based on TBP theory and used semi-structured interviews to collect information[20].

2.2 Participants

Purposive sampling method was used in this study. The sample was maximally differentiated based on different information such as gender, age, occupation, and education. No new themes emerged from the coded results when the study interviewed the 28th participant, and neither did the 29th participant. We therefore took this as data saturation[21]. Inclusion Criteria: (1) Lay rescuers with normal communication and comprehension skills; Exclusion Criteria: (1) Persons whose interview process was interrupted; (2) Persons with severe physical and psychological illnesses that prevented them from performing CPR. A total data of 29 were collected in this study. Information on all participants was presented in Table 1.

Table 1 Sociology of participant population

N	City Code	Gender	Age	Occupation	Education
1	CS	Male	30-35	Teacher （Legal Profession）	P （PhD）
2	YH	Male	20-25	Student （Economics）	U
3	K	Female	20-25	Studen (Landscape Architecture）	P
4	CZ	Female	20-25	Police	U
5	SY	Female	25-30	Teacher	U
6	CS	Male	20-25	Student （Finance）	P
7	WH	Male	25-30	Student （Design）	U
8	CS	Male	30-35	Businessmen	U
9	ZZ	Male	20-25	Student (Information and Computers）	U
10	CS	Male	16-20	Farmer	J
11	HW	Male	20-25	Student	J

				（Engineering Costs）	
12	GZ	Female	20-25	Student （International Business）	P
13	XM	Male	25-30	Teacher	P
14	CS	Male	25-30	Engineers	U
15	CS	Male	20-25	Student （Applied Psychology）	J
16	LD	Female	20-25	Self/Media	U
17	NN	Female	20-25	Student (Pharmacy)	U
18	CZ	Female	25-30	White collar	U
19	CS	Female	20-25	Student （Architectural History and Theory）	P
20	GL	Male	20-25	Student （Musicology）	U
21	KM	Female	20-25	Student （Management）	P
22	CZ	Female	25-30	Teacher	U
23	XT	Female	25-30	Student （law）	P
24	XA	Male	20-25	Student （Electronic Information）	P
25	CZ	Female	30-35	Service workers	J
26	CS	Female	30-35	Civil Servants	J
27	CS	Male	40-45	Freelance	J
28	CS	Female	25-30	Unemployed	U
29	CS	Male	20-25	Student （Psychology）	U

Abbreviations: Undergraduate=U; Postgraduate=P; Junior college=J

2.3 Process

A total of 29 respondents from different ages, occupations and regions were recruited for this study from 1/January/2022 to 20/May/2022 using online and offline purposive sampling methods. After that, they were required to give informed consent and sign the informed consent form (written or electronic), For study participants who

were minors, we obtained two-person written consent from the study participant and his/her guardian after communicating with the guardian over the phone and sending a link via the Internet. Then the researchers conducted interviews with the respondents either offline or online by video. All interviewers were systematically trained beforehand. Two researchers were present during the interview and recorded the entire process. One researcher was responsible for conducting the interview with the respondent, while the other for recording the respondent's specific facial expressions, gestures, and other important non-verbal information. Before the interview, respondents were given a detailed description of the research process; they were informed that the interview process was not usually uncomfortable and that they could pause or terminate the conversation at any time if they felt uncomfortable or tired. At the end of the interview, respondents were given a small gift worth 10 RMB. Each interview lasted approximately 20-30 minutes.

2.4 Data collection

This study was guided by the TPB. We also reviewed relevant literature and consulted experts to develop an interview outline, which was revised and discussed again by the team members after the interviews with the first two lay rescuers, so as to form a formal interview outline. See Figure 1 for details.

Figure 1 Interview outline based on the Theory of Planned Behaviour

2.5 Data analysis

The audio data will be collated and summarised within 24 hours of the interviews. Three researchers were involved in the data analysis, a professor of nursing (Z_1) and

two postgraduate nursing students (Z_2, L) trained in systematic qualitative research. The researcher (Z_2) transcribed the audio files into text. Both Z and L played back the files and modified the pauses, tone and emotional punctuation of the text. This was to minimise the loss of information caused by the transcription process. The final text was numbered; the text and audio files were imported for analysis and coding into Nvivo10, a qualitative research software that helps to collect and code audio and text files. Data uploaded to Nvivo were created as separate files and named according to their previous number. The raw data was additionally saved and recorded for later review. Independent coding was carried out by two researchers first using thematic analysis[22] and themes were compared and discussed. Then a third researcher analysed the controversial parts and drew conclusions from the raw audio material.

2.6 Credibility and rigour

The study adopted the Consolidated Criteria for Reporting Qualitative Research (COREQ)[23] to increase its credibility and rigour. Firstly, each interview was conducted in a quiet environment. Its location was determined based on each interviewee's intention to ensure that he or she felt as relaxed and comfortable as possible. Once the interviewees had stated their views, the researcher repeated the main findings and continued with further interviews. All interviewees' physical movements that might represent emotional changes were recorded. The interviewer contacted the respondent by telephone beforehand to establish their mutual trust. In addition, credibility was also established through the ongoing reading and analysis of the data. As mentioned earlier, all interviews were conducted by two researchers with experience

in qualitative research, with the same interview guide and recording equipment. Once all analyses were completed, the researchers randomly contacted three previous interviewees by telephone to feedback the results to them. This ensured the reliability and accuracy of the findings. Moreover, in terms of reflection, the research diaries consistently recorded the researchers' coding and analysis processes to examine their own conceptual perspectives, ways of summarising and generalising, and how these influenced decision-making in the qualitative research.

2.7 Ethical consideration

Informed consent was obtained from the interviewees for this study and their anonymised information is published herein. This study was approved by the Ethics Committee of Wuhan University People's Hospital. (WDRY2021-K054).

3 Results

According to the TPB, the factors that influence the public's intention to perform CPR and the conversion from intention to behavior include eleven themes and six sub-themes, as shown in Figure 2.

Figure 2 Factors affecting lay rescuers' intention to perform CPR

3.1 Attitude

3.1.1 Theme 1: Emotional resistance to the event of death

CPR is so closely associated with the word "death", which the lay rescuer is rarely confronted with. The vast majority of professional rescuers will face strong negative emotions when facing or even mentioning to a death event. These include feelings of guilt for failed rescue attempts and fear of death.

Guilt: "Because you didn't even do something correctly that caused him to die faster. This will give you a very strong sense of guilt". (13)

Fear: "I'm afraid of the whole 'death' thing, especially when it comes to things like life, which makes me feel really bad". (19)

3.1.2 Theme 2: Awareness of rewards by performing CPR

3.1.2.1 Sub-theme 2-1: Possible external rewards

Lay rescuers are encouraged to perform CPR for the potential material or reputational rewards for successfully saving a patient, which might receive from the patient's family or the community, as some participants indicated. And if the patient was good-looking, they would be more than happy to help.

"Well, if the patient is good-looking, then I'd accept to get involved in the resuscitation; and if the richest man in China falls to the ground, I'd also be very inclined to go up there and resuscitate him. I think I'd be paid a lot of money in return afterwards." (09)

3.1.2.2 Sub-theme 2-2: Fulfillment of one's intrinsic self-worth

Different cultural backgrounds have a solid and far-reaching impact on individuals. In terms of rescuing others, traditional Chinese culture positively encourages individuals, because it holds that rescuing life is the noblest thing and the obligation for everyone. Meanwhile, a participant's success in rescuing a patient can bring a strong sense of achievement and realise his or her personal value.

Sense of accomplishment: "Because I feel righteous. If I save someone, I also feel accomplished because I am still a young person with a sense of social justice" (09)

"Saving lives is a traditional virtue of the Chinese nation! As the saying goes, saving a life is better than building a seven-layer pagoda!" (02)

3.2 Perceived behavioral norms

3.2.1 Theme 3: The bystander effect

When OHCA occurs in a public place, bystanders are often more than one. The bystander effect results in a greatly reduced sense of personal responsibility. Some interviewees said that if they were alone, they would have to rescue the patients anyway. However, when there are many other bystanders at the scene, they may expect others to rescue.

"There were so many people around there. I thought it wouldn't be necessary for me to go, right?" (P23)

3.2.2 Theme 4: The encouragement from peers

Facing an OHCA event alone, individuals are prone to be overwhelmed or fearful. At this point, individuals tend to seek psychological support from those around them. Encouragement from peers can effectively alleviate their negative emotions, thus attempting to perform CPR.

"I think being with a familiar friend will give me more moral support. At least there is someone who can (stay with me). Then I can be less scared and have less worries." (24)

3.2.3 Theme 5: Motivation through personal careers

Occupations have a profound effect on people, and often similar professionals share the same personality traits. Some special professions such as police and teachers

give individuals stronger senses of justice and social responsibility, which in turn promotes them to give a helping hand to OHCA patients.

"I will definitely rush to the rescue. It's the duty of the people's police, isn't it?" (04)

3.3 Perceived behavior control

3.3.1 Theme 6: Consideration of the possible consequences to be borne

3.3.1.1 Sub-theme 6-1: Involvement in various legal disputes

The public feared being caught up in legal disputes for a variety of reasons. Participants expressed the fear that if they gave a helping hand, they might fall into unwarranted legal disputes due to factors such as the worsening of patients' condition, malicious blackmail from strangers, and inability to prove that they are not the perpetrators. Moreover, some interviewees felt that the lack of clarity of the law details and the lagging nature of legal sanctions continued to lead to their lack of trust in the law, and therefore chose to stay out of "trouble".

"Because I've seen the news before. During resuscitation, the patient's ribs or something were broken, right? What if it comes after me (muttered in a low voice)." (P28)

"Ah, because a lot of people have been scammed! It's very common in the community. That's why I dare not go near (patients)" (P17)

"But there is no detailed provision for this kind of first aid which requires more specialisation. So I don't think I have the intention to help him even if such a law is enacted. (P16)

"Serve your justice over such a long period of time would be physically and mentally exhausted." (P06)

3.3.1.2 Sub-theme 6-2: Infection with a disease

When mentioning CPR to respondents, they quickly associated it with artificial respiration and blood. They also expressed the risk of being infected with unfamiliar diseases, especially the fear of well-known blood-borne diseases such as AIDS.

"Because you know there's some (viruses and bacteria) in the blood, and (I'm) afraid there's some infectious disease or something. So I'd be more concerned about that, especially AIDS, right?" . (P08)

3.3.2 Theme 7: The dual role of public opinion pressure

This study found that with the development of technology, online public opinion is becoming a major source of social pressure. It has a dual effect on the public. A portion of the participants chose to stand by as they expressed concern that they would be condemned by public due to the patient's poor prognosis after performing CPR. Another group of participants chose to perform CPR, because they said that they might be condemned if they did not help a stranger.

"If there is no successful resuscitation, or I didn't perform CPR or something else in a right way, then it would lead to a series of negative effects on the patient's breathing. I think the public opinion pressure in this community is still quite high." (P07)

"If I wasn't there to help and it has been posted on the Internet, I would have been cursed to death" (P12)

3.3.3 Theme 8: Low self-efficacy

Self-efficacy plays a crucial role in CPR among lay rescuers. A high level of self-efficacy is one of the most important reasons for facilitating CPR performance. However, participants with low self-efficacy are often due to the misperceptions of CPR and unfamiliarity with CPR skills.

"I'll call 120, but I won't do anything else. I don't think I could do it. I heard on the news that it (CPR) takes a lot of strength and I don't think I could do it because I'm not that strong." (P25)

"I'm afraid to press the patient's chest because I don't use the skills often. So I don't know exactly how much to press it. I don't know or I'll have to ... practise a bit before I dare to save someone's life" (P11)

3.4 Actual control

3.4.1 Theme 9: CPR skill acquisition

CPR skill acquisition is important for lay rescuers to transform positive intentions of help into actual behavior. Participants expressed good intentions but also helplessness in terms of their ignorance of CPR. Their gap in skills and knowledge prevented them from ultimately performing CPR.

"The main thing is that I don't know how to do ah! If the CPR training (organisation) organises the relevant test and I pass the test, then I will go (to rescue)." (P21)

"I'll help, because I've seen CPR and all that procedures before. But I've (just) seen it all and didn't deliberately try to learn it."

3.4.2 Theme 10: Impediments/support in the field environment

3.4.2.1 Sub-theme 10-1: Support for public facilities or processes

Reliable and well-established public facilities or proven assistive processes can effectively address the practical impediments faced by the public during an OHCA event. The use of defibrillators is also a key step in improving the prognosis of cardiac arrest patients, while telephone-guided CPR effectively solves the problem of personal CPR skill deficiency.

"Because if you do this on your own ... , the failure rate is probably higher than 90 per cent. But if a professional instructs you (CPR instruction via phone), the success rate is still generally a little bit higher." (P04)

"Because this portable defibrillator is not as specialised and technically demanding as CPR skills. It is just relatively simple to operate." (P28)

3.4.2.2 Sub-theme 10-2: Reduced responsibility at special locations

When OHCA occurs in different locations, there are several factors that can influence lay rescuers to perform CPR. Bystander responsibility may be reduced under the circumstances such as the distance between the patient and the lay rescuers, the distance to the hospital, and the presence of other staff. In addition, the complexity or danger of the scene may also reduce the lay rescuer's intention to help, which is common at scenes of traumatic OHCA[24].

"I think it depends on whether he's close to me, right? If he's far from me, then maybe someone will already be helping him before I get there." (12)

"If the ambulance doesn't come, or if it's a long way (for an ambulance to the

scene), I might continue to do a little bit (of rescuing). But if it's not a very long way, I'd probably choose not to do it." (29)

"Ah, I probably wouldn't. I wouldn't. But maybe I would, because now, if I was at an underground station, I'd probably call the underground staff first." (13)

"If it's a very, very bloody scene, I think I might be shocked at first, especially if it's a car crash." (08)

3.4.3 Theme 11: Impact of patient status

When the patient was a child or a young adult, bystanders were encouraged to perform CPR, whereas the status of pregnant women and the elderly discouraged bystanders from assistance. When the patient was an acquaintance of the bystanders, we obtained two very different results. Some respondents stated that they would not hesitate to perform CPR because they were acquainted. Others said that they would have been more nervous under this circumstance; they would have preferred to wait for a professional.

"If the patient is a child, then I definitely have to rescue ah. But pregnant women? I would not dare. I do not know if I can press (her chest) ... Will it hurt the foetus in her womb ah?" (P07)

"If the patient is someone I know, I would do CPR. If he or she is unknown to me, I should ..., or ..., be afraid to do these things." (P01)

"I think the bystander would feel more guilty if CPR had been given to that acquaintance but with serious harm" (P21)

4 Discussion

Based on TPB, this study explored the factors that influence lay rescuers' intentions and conversion from intentions into behavior when facing an OHCA incident. Existing evidence showed that theory-based interventions are effective in changing public health behaviors[25] such as tobacco control, condom use, and prevention and control of HIV transmission[26-28]. And in the case of CPR, Ashish R. Panchal et al. have developed a well-established paradigm based on the TPB[9]. Our findings showed that the factors influencing the lay rescuers' intention to perform CPR have changed. Their intention have reduced due to their emotional resistance to a death event, the bystander effect, the fear of getting into legal disputes,and being infected with infectious diseases, the negative effects of public opinion pressures, and low self-efficacy. The lay rescuers' intention was prevented from converting into behavior because of the poor field environment, the lack of CPR skills, and the special status of the patient. However, their intention can be increased, to a certain extent, by the positive effects like potential rewards after rescue, peer encouragement, motivation from a particular occupation, and high opinion among the public.

The results of our study showed that the CPR training system requires further improvement. For example, the current teaching content focuses solely on the acquisition of skills, with a lack of relevant psychological education. This has resulted in a lack of rational thinking and psychological resilience to resist negative emotions when faced with OHCA incidents. This has resulted in a lack of rational thinking and psychological resilience among the public to resist negative emotions in the face of an

OHCA event. *The AHA Guidelines* presented that the rescuers in an OHCA event may suffer from severe emotional or psychological distress. Although the AHA standardized training was introduced to improve current CPR training in China, the lack of effective cultural adaptation makes it less beneficial in improving bystander CPR implementation rates[29]. Given the profound influence of social culture on individual behavioral attitudes[30], it can be concluded that the culture of death, which is closely associated with OHCA events, may acts as an impediment to CPR implementation[31]. Because for one thing, fear and avoidance of death are natural biological instincts, and for another, death in traditional Chinese culture connotes ominousness and uncleanliness. Considering the above factors, the first aid teaching content and BPR training should be closely linked to death education, and the correct standardized death education should be established to improve the public's attitudes toward death, negative emotional distress, and CPR implementation rates[32].

Individual motivation theory suggests that reward factors that satisfy an individual's needs are often an effective means of intervening in an individual's behavior[33]. The results of this study showed a similar perspective. The lay rescuers were aware that the benefits of performing CPR on OHCA patients are from both external motivations and intrinsic value satisfaction. Incentives can trigger approach or avoidance behaviour by identifying signals from potential target states[34]. Individuals make cost estimates for desired target behaviours, which promotes their first aid for OHCA patients when the estimated benefits exceed the costs[35]. At the same time, individuals have a degree of utilitarianism, which may lead them to rate the available

rewards, thus preventing them from implementing CPR[36]. This suggests that the government and society should guide the public values and cultivate individuals' characters through education or regulation, so that their utilitarian goals can be aligned with the good virtues such as "helpfulness" and "tolerance", thus promoting their rescue behaviours.

OHCA incidents occur in public places. Research has shown that they are more likely to be noticed by bystanders, thus increasing the patient's probability of being rescued[37]. However, this study found that the presence of the bystander effect[38] significantly reduced individuals' sense of responsibility, making them reluctant to take action. Meanwhile, it found that the encouragement or just accompany of familiar peers was effective in alleviating lay rescuers' negative emotions such as nervousness and anxiety, thus facilitating rescue behavior. Some special occupations may provide individuals with a more helpful spiritual core, such as police officers and teachers, whose professional beliefs may effectively increase their intention to rescue in the face of emergencies such as OHCA.

Doubts about the details of legal policies and the complexity of the legal protection have been important obstacles for lay rescuers to implement CPR, while online public opinion pressure has played a dual role to some extent. Consistent with previous research, the issue of legal protection for lay rescuers after CPR has always been of great concern to the public[39]. In this study, the participants was not sensitive to updates in the law and lacked knowledge of the relevant laws, and even the legal professionals were not familiar with the newly implemented laws. Therefore, on the

one hand, there is a need to enhance public awareness of the laws and regulations for implementing CPR; on the other hand, the existing legal system may be imperfect, and it is difficult to delineate the responsibility for bravery. These are new issues of public concern. In addition, the data showed that among 170 cases of complaints about the provision of CPR, 167 were based on the inadequate or untimely CPR by bystanders, and another 3 cases were about injuries caused by CPR[40]. Interviewers in this study also indicated that even with clear legal protections, rescuers are vulnerable to lengthy and complex legal disputes. This makes the public more fearful of the legal risks they may take after performing CPR. Therefore, the existing "Good Samaritan Law" may need to be improved with more details and simplified procedures to protect the rights of rescuers and eliminate the public's fear of taking legal liability.

The approximate 5%-55% of victims worldwide suffering online violence[41]. according to this study, have also prevented the public who concerned about online public opinion pressure from performing CPR. Meanwhile, the study found that the public may be prevented by other significant factors such as legal disputes, disease infections, blackmail, which is also consistent with previous studies[24]. The Study has also found that online public opinion plays a dual role. On the one hand, with the rapid dissemination of online information, the partial report of news, and the free expression of personal opinions, the online public opinions may turn into online violence and began to have a significant impact on public health events. However, limited research took online violence as a factor to study its impact on the public's performance of CPR, which may turn out to be an important influencing factor. On the other hand, public

opinion can exert moral pressure on individuals; their "no-rescue" behaviors may be condemned by the public online, which may encourage them to take action of performing CPR. Therefore, the government and relevant organisations should actively check the online information to promote objective descriptions of CPR-related events, and guide the dissemination of information in a correct, objective and fair manner, so as to enhance the public's implementation of CPR.

Previous studies have shown that only repeated training in CPR increased bystanders' confidence and intention to perform CPR[42-44]. However, it is difficult for the public to attend training sessions for repeated times due to factors such as inability to find a training course, time constraints, and financial burdens[45-47]. Our study also found that purely theoretical training without practice or too many participants in a single training session decreased participants' motivation. In addition, the training content did not correctly address the pros and cons of chest compression in relation to complications, which may be a disincentive for the public to be fearful of post-CPR complications such as rib fracture to patients. Future training needs to emphasise that the benefits of active treatment outweigh the dangers of CPR complications, so as to increase public self-efficacy and BCPR implementation rates.

Adequate external resources can help make CPR easier for lay rescuers. Telephone-guided CPR (T-CPR) and automated external defibrillators (AEDs) are both effective in assisting bystanders to perform CPR. Studies have shown that CPR significantly reduced the risk of panic among lay rescuers, thereby facilitating their performance of CPR[48, 49]. Respondents in our study also indicated that they would

be willing and confident to perform CPR with the help of T-CPR if they were aware of it. AEDs are effective in improving patient prognosis[50]; however, their deployment in China is insufficient and public awareness of AEDs is poor[51]. The environment in which an OHCA event occurs also determines the difficulty for bystanders to access external resources. According to previous studies, both OHCA patient's location and the scene environment influenced bystanders' intention to perform CPR[24, 52]. And this study further found that the distance between the locations of the OHCA patient and the lay rescuers affected the intention of the latter to perform CPR. Respondents indicated that if the patient was far away from their position, the closer bystander may take action first, and therefore there was no need for them to run over to the patient. Meanwhile, previous studies have found that BCPR implementation rate in developed areas and public places was higher than that in backward areas and private places due to the convenient access to external resources[53, 54]. However, this study found that when there is a hospital or a staffed location near the OHCA event, the public perceived it as more sensible to wait around the patient for the arrival of the staff or ambulance, which is most likely to cause the OHCA patient miss the "golden time" for resuscitation[55].

The impact of the patients themselves on bystanders in this study is consistent with other studies[53]. If the patient is an acquaintance of the lay rescuer, the former has a dual effect on the latter. This is encouraging for the lay rescuer to perform CPR and meanwhile makes himself/herself more nervous and unwilling to resuscitate for fear of improper handling. Pregnant women and the elderly remain barriers to CPR by lay

rescuers[44]. On the contrary, when the patient's status is of potential benefits, it increases the public's willingness to take action. Some respondents stated that compared with the potential risks, if the patient is a well-known wealthy businessman, celebrity, or good-looking person, they were willingness to rescue the patient so as to get rewards.

5 Conclusion

There are many new factors that influence lay rescuers' intention to perform CPR and their conversion from intention to behavior. Trainers should be aware of these factors and incorporate courses of psychology, death, and law into future CPR training in order to increase the public's willingness to perform CPR. Moreover, the existing CPR resources for lay rescuers should be enriched, including formulating legal policies concerning online violence, clarifying the boundaries of legal responsibility, improving the public first aid facilities such as AEDs, and making CPR skills available to all the public. In these ways, the public's willingness to implement CPR can be promoted and the barriers to practical implementation can be reduced.

6 Limitation

There are some limitations to our study. The sample was mainly centred on the 20-30 age group and may not be a representative of the true views of the whole age group. Secondly, the number of participants recruited was relatively small due to the convenience sampling method, and there is a need to expand the number of occupations, groups and regions in the future in order to understand their truer perceptions. In addition, less than 10% of respondents actually participated in or witnessed OHCA events, so the predictions of their own behaviors may not match the reality.

Conflict of interest

The authors declare that they have no conflict of interest.

Founding

This study received support from the Hunan Provincial Health Commission Scientific Research Program (Project No. D202303056656).

Author's contribution

ZHONG Zhikang: Conceptualization, Methodology, Formal Analysis, Software, Writing-Original draft preparation **LIU Liang :** Data curation, Methodology, Project administration, Supervision, Writing-Review&Editing **LI Weiwei:** Formal Analysis, Software, Writing-Original draft preparation **ZHOU Lin:** Formal Analysis, Software, Validation, Funding acquisition

References

1. Myat A, Song KJ, Rea T. Out-of-hospital cardiac arrest: current concepts. Lancet. 2018;391(10124):970-9. doi: 10.1016/s0140-6736(18)30472-0. PubMed PMID: 29536861.
2. BI S W JIA J. Scientific promotion of cardiopulmonary resuscitation (CPR) to defeat the "killer" of sudden cardiac death - The first national on-site CPR training course for teachers was held in Beijing. [J]. China Journal of Emergency Resuscitation and Disaster Medicine, 2019, 14(01): 100+102.
3. The Writing Committee of the Report on Cardiac Arrest and Cardiopulmonary Resuscitation in China. Report on Cardiac Arrest and Cardiopulmonary Resuscitation in China (2022): a Summary [J]. Chinese Circulation Journal, 2023, 38(10): 1005-1017.
4. Fukushima H, Bolstad F. Telephone CPR: Current Status, Challenges, and Future Perspectives. Open Access Emerg Med. 2020;12:193-200. Epub 20200907. doi: 10.2147/oaem.S259700. PubMed PMID: 32982493; PubMed Central PMCID: PMCPMC7490094.
5. Hou L, Wang Y, Wang W. Optimization of the Pre-Hospital Rescue System for Out-of-Hospital Cardiac Arrest in China. China CDC Wkly. 2022;4(3):52-5. doi: 10.46234/ccdcw2022.008. PubMed PMID: 35586459; PubMed Central PMCID: PMCPMC8796719.
6. Olasveengen TM, Mancini ME, Perkins GD, Avis S, Brooks S, Castrén M, et al. Adult Basic Life Support: 2020 International Consensus on Cardiopulmonary Resuscitation and Emergency Cardiovascular Care Science With Treatment Recommendations. Circulation. 2020;142(16_suppl_1):S41-s91. Epub 20201021. doi: 10.1161/cir.0000000000000892. PubMed PMID:

33084391.
7. Soar J, Böttiger BW, Carli P, Couper K, Deakin CD, Djärv T, et al. European Resuscitation Council Guidelines 2021: Adult advanced life support. Resuscitation. 2021;161:115-51. Epub 20210324. doi: 10.1016/j.resuscitation.2021.02.010. PubMed PMID: 33773825.
8. !!! INVALID CITATION !!! [8].
9. Panchal AR, Fishman J, Camp-Rogers T, Starodub R, Merchant RM. An "Intention-Focused" paradigm for improving bystander CPR performance. Resuscitation. 2015;88:48-51. Epub 20141219. doi: 10.1016/j.resuscitation.2014.12.006. PubMed PMID: 25534077; PubMed Central PMCID: PMCPMC4426863.
10. Field JM, Hazinski MF, Sayre MR, Chameides L, Schexnayder SM, Hemphill R, et al. Part 1: executive summary: 2010 American Heart Association Guidelines for Cardiopulmonary Resuscitation and Emergency Cardiovascular Care. Circulation. 2010;122(18 Suppl 3):S640-56. doi: 10.1161/circulationaha.110.970889. PubMed PMID: 20956217.
11. Link MS, Berkow LC, Kudenchuk PJ, Halperin HR, Hess EP, Moitra VK, et al. Part 7: Adult Advanced Cardiovascular Life Support: 2015 American Heart Association Guidelines Update for Cardiopulmonary Resuscitation and Emergency Cardiovascular Care. Circulation. 2015;132(18 Suppl 2):S444-64. doi: 10.1161/cir.0000000000000261. PubMed PMID: 26472995.
12. Ho AF, Sim ZJ, Shahidah N, Hao Y, Ng YY, Leong BS, et al. Barriers to dispatcher-assisted cardiopulmonary resuscitation in Singapore. Resuscitation. 2016;105:149-55. Epub 20160608. doi: 10.1016/j.resuscitation.2016.05.006. PubMed PMID: 27288652.
13. Ajzen I. From Intentions to Actions: A Theory of Planned Behavior. Springer Berlin Heidelberg. 1985.
14. Ajzen I. The theory of planned behavior: Frequently asked questions. Human Behavior and Emerging Technologies. 2020.
15. Conner M, Norman P. Predicting health behaviour: Predicting Health Behaviour; 2010.
16. Mclaws ML, Maharlouei N, Yousefi F, Askarian M. Predicting hand hygiene among Iranian health care workers using the theory of planned behavior. American journal of infection control. 2012;40(4):336-9.
17. White KM, Terry DJ, Troup C, Rempel LA, Norman P, Mummery K, et al. An extended theory of planned behavior intervention for older adults with type 2 diabetes and cardiovascular disease. Journal of Aging & Physical Activity. 2012;20(3):281.
18. ZHANG C, SHI JH. Research progress on the application of theory of planned behavior in chronic disease management [J]. Chinese Nursing Research, 2023, 37(07): 1208-1212.
19. Lee S, Vincent C. Analysis and Evaluation of the Theory of Planned Behavior. ANS Adv Nurs Sci. 2021;44(4):E127-e40. doi: 10.1097/ans.0000000000000369. PubMed PMID: 34225283.
20. Dicicco-Bloom B, Crabtree BF. The qualitative research interview. Med Educ. 2006;40(4):314-21. doi: 10.1111/j.1365-2929.2006.02418.x. PubMed PMID: 16573666.
21. Fishbein M, Ajzen I. Predicting and Changing Behavior: The Reasoned Action Approach: Prediction and Change of Health Behavior: Applying the reasoned action approach; 2010.
22. Liu M. Using an example to illustrate Colaizzi′s phenomenological data analysis method. Journal of Nursing Science. 2019;34(11):3.
23. Tong A, Sainsbury P, Craig J. Consolidated criteria for reporting qualitative research (COREQ): a 32-item checklist for interviews and focus groups. Int J Qual Health Care. 2007;19(6):349-57. Epub 20070914. doi: 10.1093/intqhc/mzm042. PubMed PMID: 17872937.

24. Lu CH, Fang PH, Lin CH. Dispatcher-assisted cardiopulmonary resuscitation for traumatic patients with out-of-hospital cardiac arrest. Scand J Trauma Resusc Emerg Med. 2019;27(1):97. Epub 20191101. doi: 10.1186/s13049-019-0679-2. PubMed PMID: 31675978; PubMed Central PMCID: PMCPMC6824105.
25. Ridde V, Pérez D, Robert E. Using implementation science theories and frameworks in global health. BMJ Glob Health. 2020;5(4):e002269. Epub 20200416. doi: 10.1136/bmjgh-2019-002269. PubMed PMID: 32377405; PubMed Central PMCID: PMCPMC7199704.
26. Levy DT, Cadham CJ, Li Y, Yuan Z, Liber AC, Oh H, et al. A Decision-Theoretic Public Health Framework for Heated Tobacco and Nicotine Vaping Products. Int J Environ Res Public Health. 2022;19(20). Epub 20221018. doi: 10.3390/ijerph192013431. PubMed PMID: 36294011; PubMed Central PMCID: PMCPMC9602493.
27. Fulmer E, Rogers T, Glasgow L, Brown S, Kuiper N. Evaluating Comprehensive State Tobacco Prevention and Control Programs Using an Outcome Indicator Framework. Health Promot Pract. 2019;20(2):214-22. Epub 20180322. doi: 10.1177/1524839918760557. PubMed PMID: 29566575; PubMed Central PMCID: PMCPMC8106980.
28. Harris J, Thaiprayoon S. Common factors in HIV/AIDS prevention success: lessons from Thailand. BMC Health Serv Res. 2022;22(1):1487. Epub 20221206. doi: 10.1186/s12913-022-08786-6. PubMed PMID: 36474252; PubMed Central PMCID: PMCPMC9727911.
29. Xu F, Zhang Y, Chen Y. Cardiopulmonary Resuscitation Training in China: Current Situation and Future Development. JAMA Cardiol. 2017;2(5):469-70. doi: 10.1001/jamacardio.2017.0035. PubMed PMID: 28297007.
30. Kitayama S, Park J. Cultural neuroscience of the self: understanding the social grounding of the brain. Soc Cogn Affect Neurosci. 2010;5(2-3):111-29. doi: 10.1093/scan/nsq052. PubMed PMID: 20592042; PubMed Central PMCID: PMCPMC2894676.
31. Chiang FM, Wang YW, Hsieh JG. How Acculturation Influences Attitudes about Advance Care Planning and End-of-Life Care among Chinese Living in Taiwan, Hong Kong, Singapore, and Australia. Healthcare (Basel). 2021;9(11). Epub 20211030. doi: 10.3390/healthcare9111477. PubMed PMID: 34828523; PubMed Central PMCID: PMCPMC8621689.
32. Testoni I, Iacona E, Corso C, Pompele S, Dal Corso L, Orkibi H, et al. Psychology Students' Perceptions of COVID-19 in a Death Education Course. Front Public Health. 2021;9:625756. Epub 20210416. doi: 10.3389/fpubh.2021.625756. PubMed PMID: 33937167; PubMed Central PMCID: PMCPMC8086793.
33. Bandhu D, Mohan MM, Nittala NAP, Jadhav P, Bhadauria A, Saxena KK. Theories of motivation: A comprehensive analysis of human behavior drivers. Acta Psychol (Amst). 2024;244:104177. Epub 20240213. doi: 10.1016/j.actpsy.2024.104177. PubMed PMID: 38354564.
34. Berridge KC, Robinson TE, Aldridge JW. Dissecting components of reward: 'liking', 'wanting', and learning. Curr Opin Pharmacol. 2009;9(1):65-73. Epub 20090121. doi: 10.1016/j.coph.2008.12.014. PubMed PMID: 19162544; PubMed Central PMCID: PMCPMC2756052.
35. Morris LS, Grehl MM, Rutter SB, Mehta M, Westwater ML. On what motivates us: a detailed review of intrinsic v. extrinsic motivation. Psychol Med. 2022;52(10):1801-16. Epub 20220707. doi: 10.1017/s0033291722001611. PubMed PMID: 35796023; PubMed Central PMCID: PMCPMC9340849.
36. Mill JS. Utilitarianism. History of Economic Thought Books. 1993;58(2):1772.
37. Kim SY, Lee SY, Kim TH, Shin SD, Song KJ, Park JH. Location of out-of-hospital cardiac arrest and the awareness time interval: a nationwide observational study. Emerg Med J. 2022;39(2):118-23.

Epub 20210623. doi: 10.1136/emermed-2020-209903. PubMed PMID: 34162629.
38. Luo Y, Sun R, Wang Y, Wei Z. Cognitive Resources:A New Perspective to Explain Social Facilitation. Journal of Psychological Science. 2021;44(5):8.
39. Mrozek K, Praclawski A. Good Samaritan Clause. Ginekol Pol. 2021;92(3):216-9. Epub 20210310. doi: 10.5603/GP.a2020.0188. PubMed PMID: 33751519.
40. Murphy TW, Windermere S, Morris T, Slish J, Holtzman L, Becker TK. Risk and ROSC - Legal implications of bystander CPR. Resuscitation. 2020;151:99-102. Epub 20200404. doi: 10.1016/j.resuscitation.2020.03.017. PubMed PMID: 32259608.
41. Mallik CI, Radwan RB. Adolescent victims of cyberbullying in Bangladesh- prevalence and relationship with psychiatric disorders. Asian J Psychiatr. 2020;48:101893. Epub 20191205. doi: 10.1016/j.ajp.2019.101893. PubMed PMID: 31865200.
42. Chen M, Wang Y, Li X, Hou L, Wang Y, Liu J, et al. Public Knowledge and Attitudes towards Bystander Cardiopulmonary Resuscitation in China. Biomed Res Int. 2017;2017:3250485. Epub 20170307. doi: 10.1155/2017/3250485. PubMed PMID: 28367441; PubMed Central PMCID: PMCPMC5359437.
43. Ballesteros-Peña S, Fernández-Aedo I, Pérez-Urdiales I, García-Azpiazu Z, Unanue-Arza S. [Knowledge and attitudes of citizens in the Basque Country (Spain) towards cardiopulmonary resuscitation and automatic external defibrillators]. Med Intensiva. 2016;40(2):75-83. Epub 20151129. doi: 10.1016/j.medin.2015.10.004. PubMed PMID: 26645945.
44. Becker TK, Gul SS, Cohen SA, Maciel CB, Baron-Lee J, Murphy TW, et al. Public perception towards bystander cardiopulmonary resuscitation. Emerg Med J. 2019;36(11):660-5. Epub 20190831. doi: 10.1136/emermed-2018-208234. PubMed PMID: 31473603.
45. Hawkes CA, Brown TP, Booth S, Fothergill RT, Siriwardena AN, Zakaria S, et al. Attitudes to Cardiopulmonary Resuscitation and Defibrillator Use: A Survey of UK Adults in 2017. Journal of the American Heart Association. 2019;(7).
46. ZHANG R Y FENG H L, MA J H, et al. Investigation on cognition and training willingness of cardiopulmonary resuscitation among the public [J]. Occup and Health, 2023, 39(10): 1359-1363. https://doi.org/10.13329/j.cnki.zyyjk.2023.0260
47. Jiang Y, Wu B, Long L, Li J, Jin X. Attitudes and willingness toward out-of-hospital cardiopulmonary resuscitation: a questionnaire study among the public trained online in China. BMJ Open. 2020;(10).
48. Moon S, Ryoo HW, Ahn JY, Lee DE, Shin SD, Park JH. Association of response time interval with neurological outcomes after out-of-hospital cardiac arrest according to bystander CPR. Am J Emerg Med. 2020;38(9):1760-6. Epub 20200602. doi: 10.1016/j.ajem.2020.05.108. PubMed PMID: 32739845.
49. Lee SY, Hong KJ, Shin SD, Ro YS, Song KJ, Park JH, et al. The effect of dispatcher-assisted cardiopulmonary resuscitation on early defibrillation and return of spontaneous circulation with survival. Resuscitation. 2019;135:21-9. Epub 20190109. doi: 10.1016/j.resuscitation.2019.01.004. PubMed PMID: 30639789.
50. Page RL. The AED in resuscitation: it's not just about the shock. Trans Am Clin Climatol Assoc. 2011;122:347-55. PubMed PMID: 21686237; PubMed Central PMCID: PMCPMC3116356.
51. HUANG M J, WANG Y J, XIONG W N, et al. An investigation of public awareness of automated external defibrillators and willingness to perform defibrillation behaviour [J]. Modern Nurse, 2023, 30(11): 114-117. https://doi.org/10.19791/j.cnki.1006-6411.2023.31.029
52. Ro YS, Shin SD, Lee YJ, Lee SC, Song KJ, Ryoo HW, et al. Effect of Dispatcher-Assisted

Cardiopulmonary Resuscitation Program and Location of Out-of-Hospital Cardiac Arrest on Survival and Neurologic Outcome. Ann Emerg Med. 2017;69(1):52-61.e1. Epub 20160921. doi: 10.1016/j.annemergmed.2016.07.028. PubMed PMID: 27665488.

53. Chen YC, Yu SH, Chen WJ, Huang LC, Chen CY, Shih HM. Dispatcher-Assisted Cardiopulmonary Resuscitation: Disparity between Urban and Rural Areas. Emerg Med Int. 2020;2020:9060472. Epub 20200601. doi: 10.1155/2020/9060472. PubMed PMID: 32566308; PubMed Central PMCID: PMCPMC7285275.

54. Li X, Zheng K, Wang J, Zhang J, Ma Q. The investigation of the awareness rate and implementation rate of emergency medical service workers about telephone-assisted cardiopulmonary resuscitation in Beijing Chinese Journal of Critical Care Medicine. 2020;40(8):4.

55. He Y, Yao P, Xiang X, Zhou T-Y, Wang Z, Cao Y, et al. Interpretation of the updates in the adults cardiopulmonary resuscitation in 2022International Consensus on Cardiopulmonary Resuscitation and Emergency Cardiovascular Care Science with Treatment Recommendations. West China Medical Journal. 2023.

Interview outline based on the Theory of Planned Behaviour

Click here to access/download;Figure;Fig 1.tiff

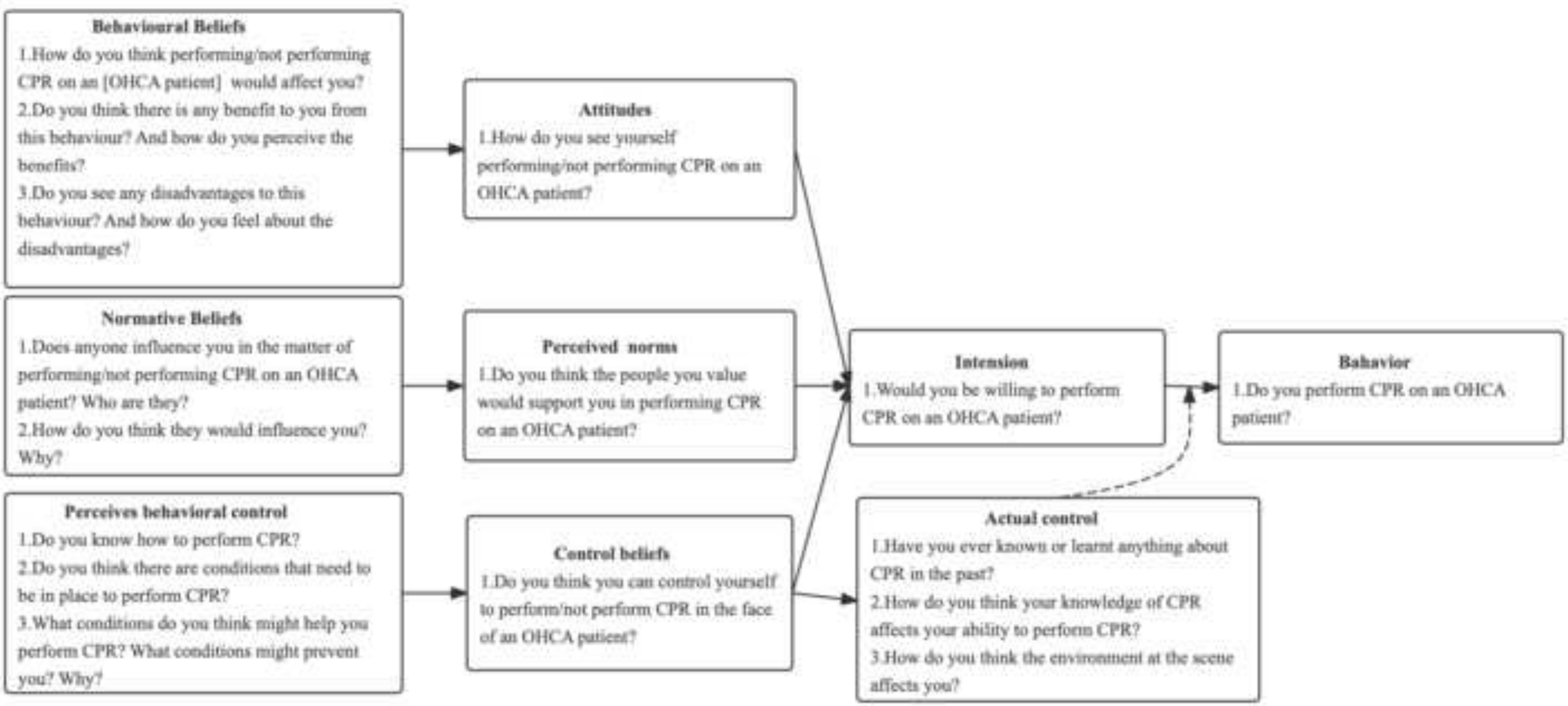

 Click here to access/download;Figure;Fig 2.tiff

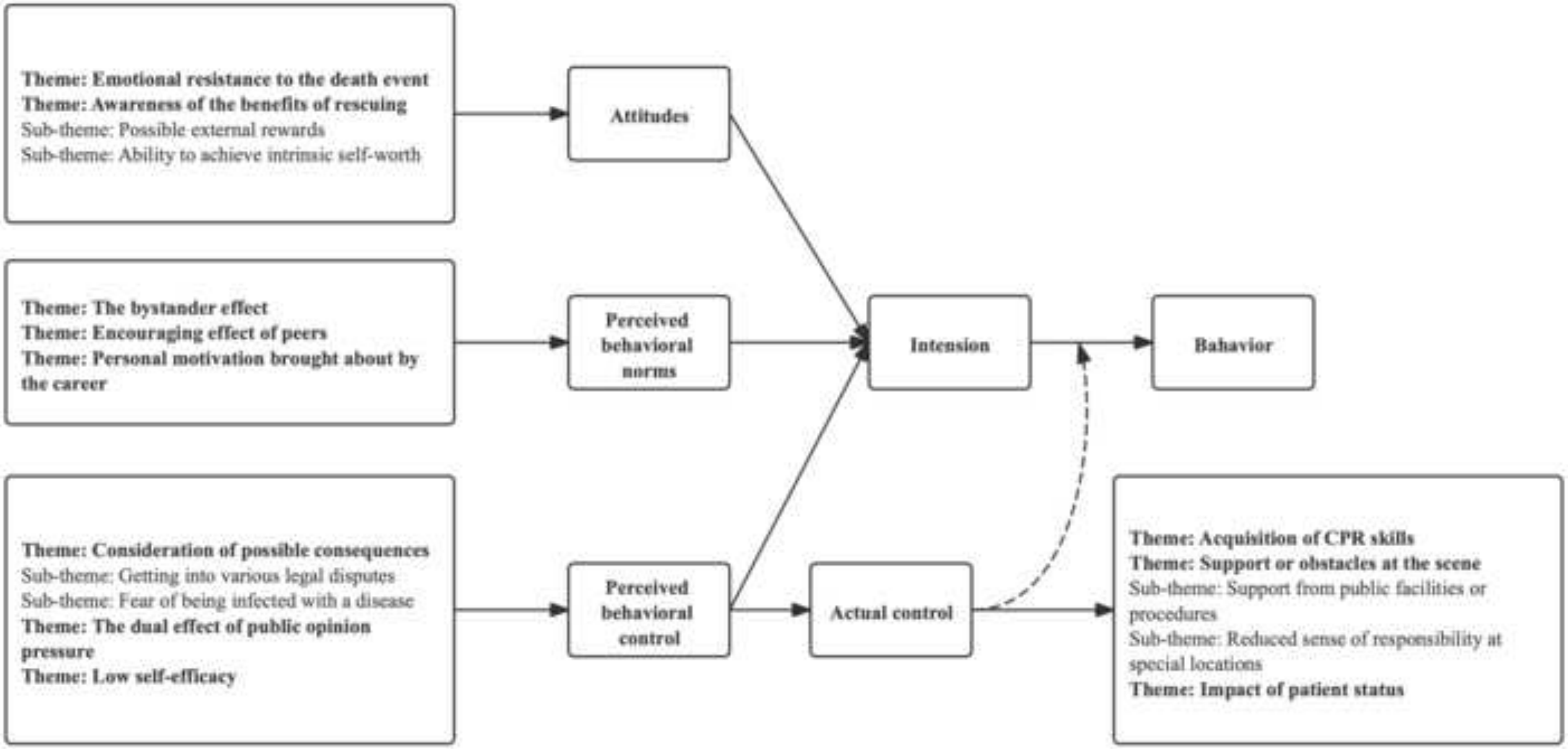

Dear Dr. Jeanelle Pauline P. Cupino

Thank you very much for your pertinent comments on the manuscript. We have carefully examined and thought about the issues raised and responded with the following comments:

1. During our evaluation of the documents provided, we noted that your ethics approval letter did not cover the entire date range for participant recruitment. Before we can proceed further with the submission, please provide the ethics approval extension document(s) for the study. If the document is in another language, please also provide an English translation.

We double-checked the time of recruitment of study participants and therefore corrected the actual time of recruitment of study participants.

2. We kindly ask that you address the discrepancy in the ethics documentation. The individual Chen Jing named as investigator on the ethics documents is not an author on the present submission. As we would generally expect the investigator named on the ethical approval to be an author on the submission, can you please provide an explanation for this situation?

The name of the main person in charge of the research project, Chen Jing, was filled in the ethical document, so the ethical approval was an ethical review of the whole project, of which the present study was a component part. Wei-Wei Li and Zhi-Kang Zhong were the participants of the project. Chen Jing's actual contribution to this part of the study was small and therefore not categorised as a lead author.

3. You indicated that you had ethical approval for your study. In your Methods section, please ensure you have also stated whether you obtained consent from parents or guardians of the minors included in the study or whether the research ethics committee or IRB specifically waived the need for their consent.

We strictly followed ethically relevant principles throughout the study, and we describe the corrections to this section in the text as follows:

For study participants who were minors, we obtained two-person written consent from the study participant and his/her guardian after communicating with the guardian over the phone and sending a link via the Internet.

4. We have noticed that you have provided two dates for recruitment (May 2022 - August 2022 and May - November 2022). Please clarify and ensure that you report in the Methods section the start and end of the recruitment period for this study (recruitment refers to when the participants were formally enrolled into the study) in DD/MM/YYYY format.

We have rechecked the full text and thank you for your comments.

5. Bias in online submission information

Thank you very much for your comments, we have re-examined some of the information in the online submission and updated some of it to improve the rigour of the study. Thank you again for your kind words!

6. As requested earlier please update the correct participants' recruitment date in DD/MM/YYYY format.

Thank you very much for your constructive comments on the article, after receiving your email, our team has further verified and asked for confirmation, thank you for waiting, we have made the corrections in the article as requested, thank you very much for your help, if you have any questions please do contact me, good luck with your work.

Kind regards,

Liang Liu
