# Supplementary material for: Factors influencing the performance of cardiopulmonary resuscitation by lay rescuers: A qualitative study based on the Theory of Planned Behavior
Source: PLoS One. 2025 Jul 23;20(7):e0327439. doi: 10.1371/journal.pone.0327439 (PMC12286394; doi:10.1371/journal.pone.0327439)
Supplement: Attachment — Submitted filename: Response to Reviewers.docx [file pone.0327439.s004.docx]

ID：PONE-D-24-25699

Title: Factors influencing the performance of cardiopulmonary resuscitation by lay rescuers: a qualitative study based on the Theory of Planned Behavior

**Dear Editors:**

Thank you for your kind letter and your careful work regarding our manuscript. We have revised the manuscript in accordance with your comments. And point-by-point responses to the comments were as follows:

**1 Please ensure that your manuscript meets PLOS ONE's style requirements, including those for file naming.**

**Response:** We sincerely thank the editors for their careful review of our manuscript. In response to the feedback provided, we have made the following adjustments: **(a)** We have reorganized the manuscript’s sections to improve its clarity and logic, ensuring its alignment with PLOS ONE’s style requirements. **(b)** All files have been renamed in accordance with PLOS ONE’s file naming requirements. We hope these changes addressed the revision requirements and contribute to a clearer presentation of our work. We look forward to your further feedback and hope that our manuscript is now fully aligned with the journal’s formatting expectations.

**2. Thank you for stating the following financial disclosure:**

“This study was supported by a scientific research project from the Hunan Provincial Health Commission (Project No. D202303056656), which was awarded the project to Ms ZHOU Lin, which provided financial support for the subsequent APC.” **(Line 15, page 26)**

Please state what role the funders took in the study.  If the funders had no role, please state: "The funders had no role in study design, data collection and analysis, decision to publish, or preparation of the manuscript."

**Response:** We sincerely thank the editors for their careful review, and we apologize for any oversight on our part. Ms. Zhou Lin, as the principal investigator, provided significant contributions to the formal analysis of the data, the interpretation of results, and the revision of the manuscript. We have now included this information in the “Author Contributions” section to accurately reflect her role in the study.

**3. We note that your Data Availability Statement is currently as follows: [All relevant data are within the manuscript and its Supporting Information files.]**

Please confirm at this time whether or not your submission contains all raw data required to replicate the results of your study. Authors must share the “minimal data set” for their submission. PLOS defines the minimal data set to consist of the data required to replicate all study findings reported in the article, as well as related metadata and methods (https://journals.plos.org/plosone/s/data-availability#loc-minimal-data-set-definition).

**Response:** We appreciate your feedback regarding the data availability statement. Thank you for your feedback regarding the data availability statement. We fully support PLOS ONE's requirements concerning data availability, as they enhance the quality of research. We have revised the data availability statement in accordance with your suggestions: All additional supporting information files are accessible through the openICPSR database at the following link: https://doi.org/10.3886/E213184V2. We hope that these modifications will better align with PLOS ONE's requirements for data availability.

**Dear reviewers:**

Thank you for your careful review and constructive suggestions regarding our manuscript. We have revised the manuscript in accordance with the comments and marked all the emendation on our revised manuscript.

Reviewer #1: The is a paper discussing the factors influencing the performance of cardiopulmonary resuscitation by lay rescuers. This topic would be very important for the practical field. The paper is well-structured, with a clear introduction, methodology, results, and discussion sections. However, some sections could benefit from more detailed subheadings to guide the reader through complex arguments.
**1. It would be very helpful for the authors to re-organise the literature review. Especially, please ensure that the theoretical framework is consistently referenced throughout the paper. This will help to tie the various sections together and reinforce the central arguments.**

**Response:** We would like to thank the Reviewer #1 for the constructive feedback. In response, we have reorganized the manuscript to improve clarity and strengthen the connections between key concepts. The following changes were made:

In response to the reviewers' comments, we made significant revisions to the literature review, streamlined the research background, and concentrated on the influencing factors (barriers, **(Line 4, page 5)**) related to the exploration of bystander cardiopulmonary resuscitation through the lens of the Theory of Planned Behavior (TPB). Additionally, we incorporated visual elements to enhance the clarity of the main ideas presented. **(Line 11, page 6)** Moreover, the theoretical framework of TPB has been consistently applied throughout the data collection and analysis process **(Line 2, page 8)** Finally, in the discussion section, we focused on the interplay between the TPB components: “Attitude”, “Subjective Norm”, and “Perceived Behavioral Control”. We restructured the discussion into three key themes based on practical considerations: Adaptation of CPR Training Strategies **(Line 12, page 18)**, Updates to Laws and Policies **(Line 4, page 20)** and Control of Real-World Environments **(Line 22 page 22)**

This structure aims to provide clearer and more actionable insights that are directly tied to the TPB framework and its practical implications.

In summary, these revisions are intended to unify the central theory of the article and strengthen the connections between the theoretical basis and the practical recommendations. By focusing on the interaction between TPB components and dividing the discussion into actionable themes, we believe the manuscript offers a clearer and more cohesive direction for future interventions.
**2. The existing theoretical framework is almost fully based on extended TPB. It would be important for the study to highlight the theoretical novelties by highlighting the difference between this work and other similar publications.**

**Response:** We sincerely thank the Reviewer #1 for the constructive comments on the article. In response to the feedback, we have revisited the existing literature and emphasized the theoretical novelties of our work. As stated in the manuscript **(Line 14, page 5)**, “It is important to note that there are currently limited studies that examine the experiences of lay rescuers perform CPR within the framework of this theory.” Most existing studies in this field have employed cross-sectional or quantitative methodologies, focusing on the validity of the TPB model in various contexts. For example:

A qualitative study by Andrews et al. (2018) explored factors influencing healthcare professionals’ use of Automated External Defibrillators (AEDs) in hospital settings, offering valuable insights into healthcare providers’ decision-making processes [Andrews et al., 2018].

Panchal et al. (2015) proposed an “Intention-Focused” paradigm for improving bystander CPR performance, but this work remains relatively broad and lacks an in-depth qualitative exploration of bystander experiences [Panchal et al., 2015].

In contrast, our study is among the few contemporary qualitative investigations that specifically examine the experiences of lay rescuers performing bystander CPR within the TPB framework. Our research bridges an important gap by exploring how TPB components—such as attitude, subjective norms, and perceived behavioral control—interact in real-world CPR situations. Furthermore, our study highlights the lived experiences of laypersons, which contrasts with prior work that has focused on more formal or institutional settings (e.g., healthcare workers). We believe that this experiential perspective offers valuable insights into the barriers and facilitators of CPR behavior among non-experts, and provides a nuanced understanding of how to design more effective interventions aimed at encouraging CPR in the general population.

**3. Please provide a more detailed background of data collection process and some more backgrounds of the 29 participants.**

**Response:** We would like to express our gratitude to the Reviewer #1 for the thorough and insightful comments regarding this section. In response, we have enhanced the details of the entire study process within the “Study Design & Participants” sections. **( Line 13, page 6)** “Interviews” **( Line 2, page 8)** and “Credibility and Rigor”. **( Line 18, page 9)** Furthermore, we have included additional demographic information about the participants, specifically regarding their prior participation in CPR training and any experiences they may have had with CPR rescue. We believe that these enhancements will further strengthen the rigor of the data.
**4. It would be important to highlight the implications of this research by combining the findings with practical policies and treatment. Highlight how the findings can be applied in real-world settings. This could involve discussing potential interventions, policy implications, or practical applications in various fields such as education, healthcare, or organizational behavior. Some interventions would be very helpful, for example novel interventions presented in “Pricing strategy for household energy-saving option (HESO): A novel option-based intervention for promoting household energy efficiency” would be a potential example.**

**Response:** Thank you for your insightful suggestion to emphasize the practical implications of our research. We have incorporated a more detailed discussion of how our findings can be applied in real-world settings. In the revised article, we have incorporated interventions that bridge research findings with practical application. When adjusting the CPR training strategy, we introduced the concept that first aid training should be integrated with death education. **(Line 3, page 19)** Furthermore, it is important to recognize that current first aid training primarily emphasizes quantity over quality, indicating that additional resources are necessary to address this issue **(Line 21, page 19)**. Regarding the updating of laws and policies, based on the research findings, we advocate for guiding the public to foster an 'altruistic' mentality while also respecting the 'self-benefiting' mentality. We suggest implementing incentive measures to enhance public enthusiasm for rescue efforts **(Line 17, page 20)**. Additionally, in light of the complexities of legal frameworks, establishing legal foundations and strengthening legal assistance may more effectively resolve the public's legal dilemmas. **(Line 16, page 21)** In response to the emergence of 'we media,' utilizing invisible tools based on AI and big data models to guide public opinion is a crucial strategy to address the interests and challenges posed by the Internet. **(Line 14, page 22)** When managing the physical environment, it is essential to acknowledge its complexity; thus, creating a more accessible first aid environment—such as telephone-based first aid and easy access to defibrillators—can significantly enhance the effectiveness of first aid interventions **(Line 16, page 23)**. Moreover, given the public's concerns regarding the first aid environment, further optimization of this environment should remain a priority moving forward. In summary, by implementing these strategies, the research findings can contribute to improving lay rescuers' effectiveness in real-world scenarios, ultimately leading to better health outcomes and increased survival rates from cardiac arrests. We hope this expanded discussion effectively addresses your recommendation and provides a clearer connection between the research findings and their practical applications.

**5. A more detailed discussion of the study’s limitations and how they might impact the findings would provide a more balanced perspective. This could include potential biases, limitations in the data collection process, and any assumptions made.**

**Response:** Thank you for highlighting the need to include a more detailed discussion of the study’s limitations. In response, we have expanded this section to provide a more balanced perspective, addressing potential biases, data collection challenges, and underlying assumptions. Specifically, the revised section now discusses the representativeness of the sample, including factors such as campus background, age, and geographical area. Furthermore, it examines the influence of cultural and environmental contexts, as well as the methods of data collection—such as the use of partially online and single semi-structured interviews—and the process through which research findings were derived **(Line 12, page 25)**.We believe this expanded discussion not only offers a more nuanced perspective on the study’s findings but also enhances the overall rigor and transparency of the research.

**6. While the paper is academically rigorous, consider simplifying some of the language to make it more accessible to a broader audience.**

**Response:** We sincerely thank the Reviewer #1 for the recognition of this study. Our team fully agrees with your perspective. To improve clarity and accessibility, we have simplified the language throughout the article and incorporated visual elements in certain sections to better illustrate key concepts. Additionally, we enlisted the assistance of native English speakers to enhance the clarity of the paper, ensuring it aligns with the journal's writing standards.

**7.Reviewer #2: This is a very interesting topic in the background where bystander CPR rates are either declining or stagnant despite major efforts. However, the language needs to be improved to effectively communicate what authors have done and found.**

**Response:** We sincerely thank the Reviewer #2 for the positive feedback and affirmation of this study. We greatly value your inquiries and, as a result, have thoroughly reviewed the entire text and made necessary revisions. Additionally, we sought the assistance of native English speakers to further refine the language, ensuring that the core ideas of the article are conveyed more effectively.
