# Supplementary material for: Factors influencing the performance of cardiopulmonary resuscitation by lay rescuers: A qualitative study based on the Theory of Planned Behavior
Source: PLoS One. 2025 Jul 23;20(7):e0327439. doi: 10.1371/journal.pone.0327439 (PMC12286394; doi:10.1371/journal.pone.0327439)
Supplement: Attachment — Submitted filename: Response_to_Reviewers_auresp_2.docx [file pone.0327439.s005.docx]

**Dear Editors**

Thank you for your thoughtful letter and the thorough review of our manuscript. We have revised the manuscript in accordance with the reviewers' comments. Below are our point-by-point responses to each of the comments:

**Reviewer #1-1:  The theory employed in this study has been widely used, especially in the relevant topics. The manuscript failed to prove its contribution to the theory and the knowledge body.**

Response to Reviewer’s Comment:

Thank you for highlighting this important aspect. We appreciate the opportunity to clarify how our study advances the Theory of Planned Behavior (TPB) and contributes to the broader body of knowledge in bystander CPR research. Specifically, our manuscript offers three distinct theoretical contributions, which we have explicitly articulated in the revised Discussion section:

1. **Integration of Emotional and Cultural Factors into TPB’s Attitude Dimension** (page 20, line 2): Our study extends the TPB’s attitude dimension by incorporating emotional and cultural contexts, thereby offering deeper insights into the motivational factors that influence bystanders’ CPR decisions.
2. **Contextual Risks and Dynamic Control: Reconstructing TPB’s Perceived Behavioral Control (PBC) Dimension** (page 21, line 17): We address limitations in the traditional conceptualization of PBC by emphasizing the dynamic nature of perceived control, particularly how situational risks and contextual variables significantly affect perceived control. This enhances the predictive accuracy and practical applicability of the TPB in emergency situations.
3. **Normative Conflicts and Multistage Intention Enactment: Reconstructing TPB’s Subjective Norm and Intention-Behavior Pathways** (page 23, line 14): Our study identifies and addresses critical gaps related to normative conflicts, emphasizing the complex, multistage process linking intention formation and behavioral enactment. This refined perspective recognizes that normative pressures and social conflicts dynamically shape bystanders’ intentions and subsequent behaviors.

Furthermore, our study proposes a dual-path strategy to enhance bystander CPR effectiveness. By combining emotionally adaptive training with systemic empowerment, this strategy directly addresses emotional barriers and contextual challenges, transforming hesitant bystanders into confident responders (page 24, line 12).

Through the integration of cultural and contextual nuances into the TPB framework, our research not only advances theoretical development but also lays a robust foundation for designing cross-cultural CPR interventions. The revised Discussion section (pages 19–25) explicitly delineates these theoretical advancements and their broader implications for future research.

Thank you again for your valuable feedback, which helped us clarify the theoretical contributions and implications for CPR training.

**Reviewer #1-2: It is believed that the sample is insufficient to support the findings and statements, and the revised version does not provide adequate discussion or evidence to address this.**

Response to Reviewer’s Comment:

Thank you for highlighting the concern regarding the sample size and the sufficiency of evidence. We recognize that sample size is a critical consideration in qualitative research. Below, we clarify our methodological approach and demonstrate how the revised manuscript strengthens the discussion on limitations and generalizability.

1. **Sample Adequacy in Qualitative Research:**

Our study employed purposeful sampling, guided by the principle of topic saturation, which is widely accepted in qualitative research (Birks & Mills, 2015; Olshansky, 2015; Given, 2016). We closely monitored saturation, noting that no new themes emerged after the 26th participant. To confirm redundancy, we interviewed an additional three participants, bringing the total to 29. This approach aligns with the recommendations of Hennink et al. (2017), who emphasize the in-depth exploration of complex psychosocial phenomena rather than statistical quantification.

While the sample size (N=29) may seem modest, it is consistent with best practices in qualitative research, which prioritize depth of understanding over sample size. In qualitative studies focused on understanding nuanced experiences, a smaller sample is often sufficient to reach saturation and generate meaningful insights.

2. **Addressing Generalizability and Limitations:**

To improve transparency, we have added a "Limitations" section (page 26, line 13), where we discuss potential limitations and the generalizability of our findings:

- "Although this study provides valuable insights, several limitations should be noted. First, while purposive sampling aimed to capture a diverse range of geographical and occupational backgrounds, more than 60% of participants were university-affiliated (students and faculty), which may skew the findings toward the perspectives of educated, urban populations. Second, the sample was predominantly composed of younger adults (ages 20-30), which may limit the applicability of our findings to older populations or adolescents. Finally, with only one rural participant, there is a risk of selection bias, which may affect the generalizability of our findings to rural communities."

We trust that this revised discussion clarifies our methodological choices and addresses the study’s limitations, thereby strengthening the transparency and rigor of our research.

**Reviewer #3-1：How were the participants recruited? Social media, personal connections, some organizations/networks?**

Response to Reviewer’s Comment:

Thank you for raising this important question. We describe our two-fold participant recruitment approach in the methodology section (Page 7, Line 9). First, we initiated recruitment through the researchers' personal networks to establish core participants. Subsequently, a snowball sampling technique was employed, where existing participants helped identify additional qualified candidates who met our diversity criteria across gender, age, occupation, and education levels. This strategy effectively balanced accessibility with our commitment to demographic variety while maintaining ethical recruitment standards.

**Reviewer #3-2：What do you mean by ‘normal communication and comprehension skills’? Has everyone spoken Mandarin? Was the interpreter involved if otherwise?**

Response to Reviewer’s Comment:

Thank you for prompting us to elaborate on this critical methodological aspect. The requirement of "normal communication and comprehension skills" involved a dual validation process:

**Communication competence** was assessed through:

- **Mandarin fluency verification**: All participants completed a 5-minute unstructured conversation during recruitment screening to evaluate spontaneous speech clarity and dialect neutrality.
- **Contextual responsiveness**: Ability to maintain topic relevance and demonstrate reciprocal dialogue patterns during preliminary discussions.

**Comprehension capacity** was operationalized as:

- Consistent logical coherence in responding to multi-layered interview questions.

As explicitly stated in our inclusion criteria (Page 7, Line 17), Mandarin proficiency was a prerequisite, with the following safeguards:

- **Pre-screening filter**: Self-reported Mandarin as the primary daily language (>5 years of continuous use).

**No interpreters were employed.**

**Reviewer #3-3：What do you mean by ‘interruptions’? Any interruptions? For how long?**

Response to Reviewer’s Comment:

Thank you for allowing us to clarify this procedural detail. In this context, interruptions were operationally defined as unplanned discontinuities that:

Originated from participants (e.g., urgent personal obligations, emotional instability)

Persisted beyond 10 minutes despite researcher efforts to resume

Fundamentally disrupted the interview's narrative flow (assessed via post-interview audio analysis)

We implemented a tiered response protocol:

<5 min pauses: Documented but permitted continuation

5-10 min interruptions: Triggered protocol review with optional rescheduling

>10 min discontinuities: Systematically excluded per pre-defined criteria (Page 7, Line 18)

Notably, technical interruptions (e.g., internet instability) were handled through real-time troubleshooting before applying exclusion measures. All exclusion decisions underwent dual verification by two researchers to prevent arbitrary implementation.

**Reviewer #3-4：Table 1. Why did you use age ranges instead of actual values? Some meaningful age categories, such as ‘young adults’, etc, with percentages would describe the population better. The age range is 16-45, with only one minor and one in the range of 40-45 – doesn’t look that diverse in terms of age.**

Response to Reviewer Comments:

We sincerely thank the reviewer for the valuable comments regarding our age grouping and the diversity of the sample. In response to your concerns about the use of age ranges and the limited age diversity, we provide the following explanations and revisions:

1. **Rationale for Using Age Ranges**
   In the initial design of our study, we adopted age ranges based on common practices in related fields to simplify data presentation and analysis. Moreover, given the limited sample size, further subdividing age groups could have led to insufficient numbers in each subgroup, thus reducing statistical power. Nonetheless, we acknowledge that more detailed age categories, including percentages, would enhance transparency and better meet readers’ expectations.
2. **Acknowledgement of Limitations**
   As the reviewer pointed out, the current sample mainly covers the age range 16-45 years, with few participants under 18 and in the 40-45 bracket, resulting in limited age diversity. We have explicitly discussed this limitation in the revised manuscript’s “Limitations” section. Specifically, we noted that the age distribution is predominantly weighted toward younger adults (20–30 years), which restricts insights into decision-making among adolescents and older adults. This limitation reflects the challenges of recruiting a fully age-diverse sample, especially under the constraints of the COVID-19 pandemic and available recruitment channels.
3. **Revisions and Improvements**
   To improve transparency, we have refined the age classification in the revised manuscript by adding two groups: Young Adults (YA, 16-25 years) and Middle-aged Adults (MA, 26-45 years). We have also updated the relevant table to include both the number and percentage of participants in each group to provide a clearer picture of the age distribution.
4. **Explanation of Age Diversity and Future Directions**
   The limited age diversity observed is largely due to recruitment challenges, including reliance on online interviews and purposive sampling during the pandemic, which may have excluded older adults and minors. Future research will aim to broaden recruitment strategies to include more minors and participants over 45 years old. Additionally, expanding the geographical and demographic scope will help generalize findings across different age groups and communities. We also acknowledge that the qualitative design prioritizes depth over breadth, and future quantitative studies could validate these findings by correlating age-related themes with behavioral outcomes.

We appreciate the reviewer’s insightful comments, which have greatly contributed to enhancing the rigor and clarity of our manuscript.

**Reviewer #3-5：****Participant 18 (White Collar) and Participant 27 (Freelance) – what are actual specialties of these people?**

Response to Reviewer Comments:

Thank you for your query. We have verified the backgrounds of the participants in question:

- Participant 18 (originally labeled "White Collar"):
  Actual specialty: Running an online shop (e-commerce operations).
  Clarification: While initially categorized broadly as "white-collar," his specific expertise lies in managing digital storefronts, inventory, and online sales.
- Participant 27 (originally labeled "Freelance"):
  Actual specialty: Chronic hemodialysis patient.
  Clarification: His "freelance" label referred to incidental work arrangements, but his core identity relevant to this study is his lived experience as a long-term dialysis patient.

**Reviewer #3-6：Interviews. Have you developed your own interview or adapted one based on literature review. If yes, how did you adapt and translate it from Chinese to English and vice versa? How was the quality check performed to make sure that the wording in Chinese is exactly how you intended to sound and does not prompt the respondents?** **How was the interview revised and refined?**

Response to Reviewer

Thank you for your valuable feedback on our interview methodology. We appreciate the opportunity to clarify our approach:

1. **Interview Development and Cross-Language Adaptation**

We designed a semi-structured interview guide, drawing upon both Chinese and English literature to ensure theoretical rigor and cultural relevance. The adaptation process followed these steps:

a. Bilingual Literature Synthesis:

- Conceptual Alignment: For terms or constructs with unclear or ambiguous cross-cultural equivalence, we consulted domain experts to ensure accurate conceptual representation in both languages. Additionally, bilingual scholars were engaged to resolve any translation discrepancies.

b. Translation Protocol:

- Forward-Backward Translation: A bilingual researcher translated the interview guide from Chinese to English, focusing on conceptual meaning rather than literal translation. A second independent translator performed a back-translation into Chinese. Discrepancies between the original and back-translated versions were thoroughly discussed and resolved by the research team.
- Cultural Calibration: Native speakers of both English and Mandarin reviewed the phrasing to ensure linguistic naturalness, cultural appropriateness, and neutrality.

2. **Quality Assurance Mechanisms**

Several strategies were employed to ensure linguistic accuracy and minimize response bias:

a. Cognitive Pretesting:

- Participant Paraphrasing: During the pilot phase, participants were asked to paraphrase interview questions to confirm clear understanding of their intent and meaning.
- Nonverbal Cue Monitoring: Interviewers monitored participants’ nonverbal cues (e.g., pauses, signs of confusion) to identify areas requiring clarification or rephrasing.

b. Neutrality Audits:

- Review of Question Language: Each question was reviewed to eliminate assumptions and prevent leading or biased language.
- Member Checking: Post-interview, participants confirmed the accuracy of their responses, ensuring the clarity and neutrality of the questions.

3. **Iterative Refinement During Data Collection**

The interview guide was continuously refined based on feedback obtained throughout data collection:

- Early-Interview Adjustments: Following interviews with the first 3-5 participants, any confusing or inconsistently interpreted questions were revised.
- Emergent Theme Integration: New themes identified during interviews led to adjustments in the guide, incorporating probes to explore these topics more thoroughly.

4. **Validation of Final Instrument**
   - Thematic Saturation: Data collection continued until no new themes emerged, confirming the adequacy and comprehensiveness of the interview guide.
   - Cross-Language Consistency: Final translations were tested with bilingual participants to ensure equivalence and accuracy between languages.

These methods ensured that our interview instrument was both culturally sensitive and methodologically rigorous. We also took proactive steps to avoid bias and leading questions in our design. We hope this detailed explanation addresses your concerns, and we are happy to provide further documentation if needed.

**Reviewer #3-6：I found only one minor in the table (Participant 10). What about the second minor participant?**
Response to Reviewer

We appreciate the reviewer’s feedback regarding the participants. After reviewing our records, we confirm that only one minor (Participant 10) was included in the study. To ensure ethical compliance and respect for the participant's decision-making capacity, informed consent was obtained from both the minor and their primary guardian.
**Reviewer #3-7：Could you submit the filled COREQ checklist?**
Response to Reviewer

Thank you for the opportunity to further refine and optimize the structure of our research. Your feedback has been invaluable and has guided us in presenting our work in a more rigorous and comprehensive manner. In response to the COREQ checklist you referenced, we have carefully revised and supplemented the manuscript in accordance with each item. The completed checklist has been uploaded as a separate attachment for your reference.
**Reviewer #3-8：Propaganda can be felt on social media. Algorhythms might also prevent the general population without specific interest in watching such content.**

Response to Reviewer

We appreciate the reviewer’s comment that prompted us to clarify this point. It is important to acknowledge the dual nature of algorithm-driven content dissemination. Consequently, we have updated the discussion to emphasize that effective communication strategies require the continuous adaptation of both content and delivery methods. As demonstrated in our revised analysis:

"Platforms such as TikTok and WeChat enhance prosocial behaviors by incentivizing participation through gamified rewards, thereby motivating bystander intervention during emergencies." (Page 25, Line 11)

Conversely, we argue that dynamic content updates not only expand reach among disengaged audiences but also deepen engagement for users sensitive to specific topics. Additionally, sustained exposure through algorithmic amplification remains critical for maximizing impact.

**Reviewer #3-9：What to do with pregnant and elderly people then?**
Response to Reviewer

We are grateful for the opportunity to address this critical issue regarding vulnerable populations, particularly in light of the challenges bystanders face when performing CPR due to physical and skill limitations. Vulnerable groups such as pregnant women, the elderly, and young children encounter significant difficulties during emergency interventions.

Our analysis, grounded in the theoretical framework of Perceived Behavioral Control (PBC) from the Theory of Planned Behavior (TPB), reveals the following:

"Contextual risks and dynamic control: The availability of external resources significantly moderates both risk perception and behavioral responses. Specifically, accessible automated external defibrillators (AEDs) and telephone-assisted cardiopulmonary resuscitation (T-CPR) reduce reliance on individual competencies by transferring life-saving capabilities to systematic support mechanisms."

We propose that enhancing the availability of external resources can reduce the capability-behavior gap, thereby addressing the challenges faced in rescue efforts for vulnerable populations through:

- Skill-transfer mechanisms (e.g., real-time guidance via T-CPR)
- Decision-support tools (e.g., automated AED protocols)

**Reviewer #3-10：Please, cite AHA Guidelines**

Response to Reviewer

We thank the editors for their valuable suggestions. In response, we have updated the references accordingly. Specifically, the following reference has been revised:

Panchal AR, Bartos JA, Cabañas JG, Donnino MW, Drennan IR, Hirsch KG, et al. Part 3: Adult Basic and Advanced Life Support: 2020 American Heart Association Guidelines for Cardiopulmonary Resuscitation and Emergency Cardiovascular Care. *Circulation*. 2020;142(16_suppl_2):S366-S468. Published October 21, 2020. doi: 10.1161/cir.0000000000000916. PubMed PMID: 33081529.

**Reviewer #3-11：Sometimes you mention (P13), and sometimes just put numbers. Please, be consistent – it might be a bit confusing. First I thought it was a citation.**

Response to Reviewer

We sincerely appreciate the reviewer’s thorough feedback. Regarding the two critical points raised:

1. **Participant Reference Consistency (Page 11, Line 4):**
   We apologize for the inconsistency in notation. All participant references have been standardized to the format "Participant XX" (e.g., Participant 13). This revision eliminates any potential confusion with citation markers.

**Reviewer #3-12：Please, be more cautious about the interpretation of results. Action might be different from concerns and worries, especially without actual experience.**

Response to Reviewer

We sincerely appreciate the reviewers' constructive suggestions. In response to the request for cautious interpretation, we have made the following revisions:

1. **Enhanced Analytical Rigor:**
   The results have been contextualized within the theoretical framework and subjected to structured expert validation through multidisciplinary group discussions, including emergency physicians and psychologists.
2. **Explicit Limitations:**
   We have expanded the limitations section to include the following:
   The reliance on online interviews due to COVID-19 restrictions may have limited the researchers' ability to fully observe nonverbal cues or contextual nuances typically accessible in face-to-face interactions. Additionally, the lack of CPR training among a subset of participants (exact proportion unspecified) may confound the interpretation of their self-efficacy and behavioral intentions.
3. **Theoretical Integration:**
   To address multifactorial decision-making, we propose the following theoretical model:
   "Normative Conflicts and Multistage Intention Enactment: A Reconstruction of TPB's Intention-Behavior Pathways."
