# Supplementary material for: Factors influencing the performance of cardiopulmonary resuscitation by lay rescuers: A qualitative study based on the Theory of Planned Behavior
Source: PLoS One. 2025 Jul 23;20(7):e0327439. doi: 10.1371/journal.pone.0327439 (PMC12286394; doi:10.1371/journal.pone.0327439)
Supplement: Attachment — Submitted filename: Response.docx [file pone.0327439.s006.docx]

Dear Dr. Jeanelle Pauline P. Cupino

Thank you very much for your pertinent comments on the manuscript. We have carefully examined and thought about the issues raised and responded with the following comments:

1. During our evaluation of the documents provided, we noted that your ethics approval letter did not cover the entire date range for participant recruitment. Before we can proceed further with the submission, please provide the ethics approval extension document(s) for the study. If the document is in another language, please also provide an English translation.

We double-checked the time of recruitment of study participants and therefore corrected the actual time of recruitment of study participants.

2. We kindly ask that you address the discrepancy in the ethics documentation. The individual Chen Jing named as investigator on the ethics documents is not an author on the present submission. As we would generally expect the investigator named on the ethical approval to be an author on the submission, can you please provide an explanation for this situation?

The name of the main person in charge of the research project, Chen Jing, was filled in the ethical document, so the ethical approval was an ethical review of the whole project, of which the present study was a component part. Wei-Wei Li and Zhi-Kang Zhong were the participants of the project. Chen Jing's actual contribution to this part of the study was small and therefore not categorised as a lead author.

3. You indicated that you had ethical approval for your study. In your Methods section, please ensure you have also stated whether you obtained consent from parents or guardians of the minors included in the study or whether the research ethics committee or IRB specifically waived the need for their consent.

We strictly followed ethically relevant principles throughout the study, and we describe the corrections to this section in the text as follows:

*For study participants who were minors, we obtained two-person written consent from the study participant and his/her guardian after communicating with the guardian over the phone and sending a link via the Internet.*

4. We have noticed that you have provided two dates for recruitment (May 2022 - August 2022 and May - November 2022). Please clarify and ensure that you report in the Methods section the start and end of the recruitment period for this study (recruitment refers to when the participants were formally enrolled into the study) in DD/MM/YYYY format.

We have rechecked the full text and thank you for your comments.

5. Bias in online submission information

Thank you very much for your comments, we have re-examined some of the information in the online submission and updated some of it to improve the rigour of the study. Thank you again for your kind words!

6. As requested earlier please update the correct participants' recruitment date in DD/MM/YYYY format.

Thank you very much for your constructive comments on the article, after receiving your email, our team has further verified and asked for confirmation, thank you for waiting, we have made the corrections in the article as requested, thank you very much for your help, if you have any questions please do contact me, good luck with your work.

Kind regards,

Liang Liu
